# Pyrolysis Kinetic Behaviour of Glass Fibre-Reinforced Epoxy Resin Composites Using Linear and Nonlinear Isoconversional Methods

**DOI:** 10.3390/polym13101543

**Published:** 2021-05-11

**Authors:** Samy Yousef, Justas Eimontas, Nerijus Striūgas, Marius Praspaliauskas, Mohammed Ali Abdelnaby

**Affiliations:** 1 Department of Production Engineering, Faculty of Mechanical Engineering and Design, Kaunas University of Technology, LT-51424 Kaunas, Lithuania; 2 Department of Materials Science, South Ural State University, Lenin Prospect 76, 454080 Chelyabinsk, Russia; 3 Laboratory of Combustion Processes, Lithuanian Energy Institute, Breslaujos 3, LT-44403 Kaunas, Lithuania; justas.eimontas@lei.lt (J.E.); Nerijus.Striugas@lei.lt (N.S.); 4 Laboratory of Heat Equipment Research and Testing, Lithuanian Energy Institute, Breslaujos 3, LT-44403 Kaunas, Lithuania; marius.praspaliauskas@lei.lt; 5 Department of Production Engineering and Printing Technology, Akhbar Elyom Academy, 6th of October, 12566, Egypt; Muhmmad.aly@akhbaracademy.edu.eg

**Keywords:** glass fibre-reinforced epoxy resin composites, pyrolysis, TG-FTIR-GC–MS analysis, pyrolysis kinetic, mechanical pre-treatment

## Abstract

Due to the increasing demand for glass fibre-reinforced epoxy resin composites (GFRC), huge amounts of GFRC waste are produced annually in different sizes and shapes, which may affect its thermal and chemical decomposition using pyrolysis technology. In this context, this research aims to study the effect of mechanical pre-treatment on the pyrolysis behaviour of GFRC and its pyrolysis kinetic. The experiments were started with the fabrication of GFRC panels using the vacuum-assisted resin transfer method followed by crushing the prepared panels using ball milling, thus preparing the milled GFRC with uniform shape and size. The elemental, proximate, and morphology properties of the panels and milled GFRC were studied. The thermal and chemical decomposition of the milled GFRC was studied using thermogravimetric coupled with Fourier-transform infrared spectroscopy (TG-FTIR) at different heating rates. Meanwhile, the volatile products were examined using TG coupled with gas chromatography–mass spectrometry (GC-MS). The TG-FTIR and TG-GC-MS experiments were performed separately. Linear (Kissinger–Akahira–Sunose (KAS), Flynn–Wall–Ozawa (FWO), and Friedman) and nonlinear (Vyazovkin and Cai) isoconversional methods were used to determine the pyrolysis kinetic of the milled GFRC based on thermogravimetry and differential thermal gravimetry (TG/DTG). In addition, the TG/DTG data of the milled GFRC were fitting using the distributed activation energy model and the independent parallel reactions kinetic model. The TG results showed that GFRC can decompose in three stages, and the main decomposition is located in the range 256–500 °C. On the other hand, aromatic benzene and a C-H bond were the major functional groups in the released volatile components in FTIR spectra, while phenol (27%), phenol,4-(1-methylethyl) (40%), and p-isopropenylphenol (34%) were the major compounds in GC-MS analysis. Whereas, the kinetic results showed that both isoconversional methods can be used to determine activation energies, which were estimated 165 KJ/mol (KAS), 193 KJ/mol (FWO), 180 KJ/mol (Friedman), 177 KJ/mol (Vyazovkin), and 174 KJ/mol (Cai).

## 1. Introduction

Glass fibre-reinforced epoxy resin composites (GFRC) is a well-established and essential material in the manufacture of aircraft, vehicle, and infrastructure structures [1,2]. It has wide applications in defense, electronics, renewable energy, etc. due to its outstanding physical properties including light weight and high chemical, mechanical, and thermal durability [3,4,5]. According to recent studies, the market for fibre-reinforced composites in the USA has reached $12 billion in 2020 with an expected annual growth rate of 6.6% due to its adaptation in many modern applications such as wind energy [6,7,8]. This heavy use has lead to producing a huge quantitiy of GFRC waste on a regular and increasing basis in the world [9]. The aircraft, wind energy, and electronic (waste printed circuit board) sector is the largest contributor to this share of production [9,10,11,12]. Usually, GFRC waste is composed of several layers of glass fibre collected together using resin. In addition, this type of waste can be classified based on the type of resins into thermosets (epoxy resin) and thermoplastic (acrylic poly-methyl methacrylate (PMMA)) [6,13]. In some applications, this composition is presented as a mixture with copper and other mineral layers for electrical conductivity [14,15], which are classified as heavy metals and polluting elements for soil and groundwater, as well as resins that are classified as toxic materials [16].

Therefore, it must be disposed of safely and no longer disposed by traditional methods, bad burial, burning, or throwing of waste, which are the most popular disposal methods adopted by composite industries [17] to avoid the accumulation of waste and the resulting obstacles. In addition, it can also help keep up with this strong demand for virgin glass fibres [18]. Despite all these defects, GFRC waste has a high economic value for these compounds if these materials (glass fibre and epoxy resin) are recovered in a cost-effective way without causing negative environmental effects [19]. All these factors have led to an increase in awareness about ways to dispose of such waste, where the mechanical, chemical, and thermal processes were adapted to extract the fibre and epoxy in the form of fillers or petroleum products [20,21]. Usually, mechanical treatment is used for size reduction, and converted GFRC waste into fine particles (fibres mixed with resin) in micro-size can be used as filler material in concrete, asphalt, wood, plastic, etc. [22,23,24]. In order to remove resin from the milled fibre particles, chemical treatment using organic solvents was used to dissolve epoxy resin [25], while epoxy resin fraction can be recovered from the organic solutions using a rotary evaporator. Although both processes succeeded in achieving the specific goal, some limitations have been appearing such as the need for a lot of chemicals, power consumption, etc., which makes it hard to apply for industrial scale [9].

Therefore, a pyrolysis process was used to decompose GFRC into energy products (e.g., gas, oil, and char mixed with short glass fibres) with a high calorific value in a wire-mesh reactor [26]. In addition, the thermal and chemical decomposition of GFRC was studied using thermogravimetric coupled with Fourier transform infrared spectroscopy (TG-FTIR) [27,28]. In addition, the kinetic parameters of GFRC pyrolysis were determined using different methods, including linear isoconversional methods (Kissinger–Akahira–Sunose (KAS), Flynn–Wall–Ozawa (FWO), and Friedman) and nonlinear isoconversional method (e.g., Vyazovkin). It is worth mentioning that the experiments in these studies were performed on GFRC waste in the form of a by-product collected from acid solution storage tank manufacturing factory without specific composition [27]. In another study the experiments were performed on 67% E-type glass fibre and 6509 epoxy resin without giving any attached to the composition of formulating products using GC analysis [28]. As shown, these studies did not take into account the size of waste and pre-treatment using mechanical processes, even though these practices are a necessary part of such treatments [29,30].

In this context, this research aims to study the effect of pre-treatments on the pyrolysis of GFRC. As shown, the pyrolysis experiments were performed on feedstock that has a different structure and is not similar to the commercial products. In order to put the layer of pyrolysis of GFRC and understand better its thermal and chemical decomposition and its pyrolysis kinetic parameters, this work aims to study the pyrolysis behavior of GFRC using TG-FTIR and TG-GC-MS measurements (separately). In addition, the kinetic pyrolysis of GFRC was studied using linear and nonlinear isoconversional methods. Finally, the TG curves were plotting using the distributed activation energy model (DAEM) and Independent Parallel Reactions Kinetic Model (IPR) [31,32].

## 2. Experimental

### 2.1. Materials and Design of the Research Experiments

**Figure 1:**
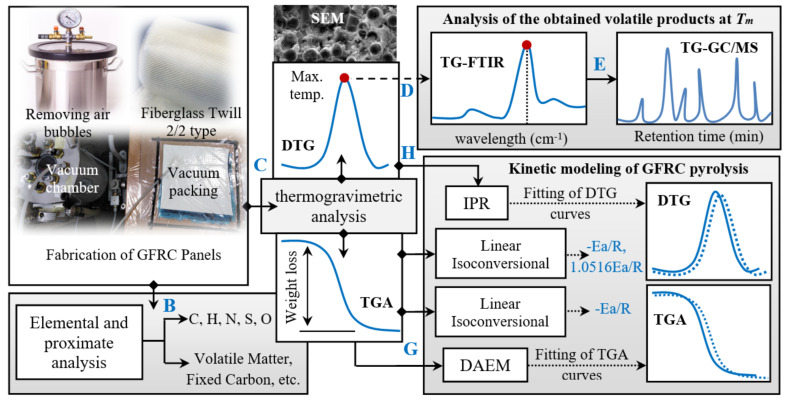
The layout of the present research.

Glass fabric Panda^TM^ (Weave: Twill 2/2 type and weight: 163 g/m^2^) was purchased from R&G Faserverbundwerkstoffe GmbH, Germany. Epoxy resin and its hardener (EPIKOTE Resin MGS^®^ RIMR 135 and EPIKURE Curing Agent MGS^®^ RIMH 1366) were supplied by Momentive. Other chemicals used in this research were purchased from Sigma-Aldrich, and the gases used in pyrolysis experiments were supplied by Lithuanian Energy Institute. Figure 1 shows the flowchart of the experiments and analysis in the present research. As shown, the research was designed in five stepes: (a) preparation of fibreglass/epoxy laminate panels using vacuum-assisted resin transfer method, (b) characterisation of GFRC, using elemental, proximate, and composition analysis, (c) study of the thermal decomption of GFRC using thermogravimetric analysis, (d,e) study of the chemical compounds of the obtained volatile compounds using FTIR and GC/MS meareaments, (f,g) study of pyrolysis kinetic parameters of GFRC using linear and nonlinear isoconversional methods, and (h,i) fitting the TG-DTG exponential data of GFRC waste using DAEM and IPR models, respectively. All these phases and their optimum conditions are illustrated in details in the following sections.

### 2.2. Preparation of GFRC Panel

In order to prepare the fibreglass/epoxy laminate panel (with a surface area of 100 cm^2^ and thickness 1 mm), four Glass fabric Panda^TM^ sheets (with nominal size of 10 × 10 cm) were cut from the supplied fabric roller; then, they were adhesively stuck together with epoxy/hardener solution using the vacuum-assisted resin transfer method. After that, the fabricated panels were post-cured using an infrared lamp at 75 °C for 8 h, exposed to the main curing treatment in oven at 85 °C for 6 h for achieving of the cross-linking of polymer structure and the homogeneity, thus preparing the final GFRC panel [1,2]. Finally, the cured panel was cut into small pieces (100 mm^2^) and then crushed into fine particles to minimise mass and heat-transfer resistances during the thermal decomposition using TGA analysis [33]. The milling process was performed using ball milling (mill model Fritsch P-5) at the frequency of 20 Hz for 30 min grinding, thus preparing a powder having a high degree of fineness from the crushed GFRC [25].

### 2.3. Characterisation of the Milled GFRP

The morphology of the obtained GFRC panel and the crushed GFRP samples were observed using Scanning Electron Microscopy (SEM) and Metallurgical Microscope, receptivity. The carbon (C), nitrogen (N), hydrogen (H), and sulphur (S) content in the milled GFRP sample was measured with an Elemental Analyser (Perkin Elmer 2400 CHN), while the proximate analysis was used to determine the amount of moisture, volatile matter, and ash in the milled GFRP sample according to ASTM standard methods (E1756-01, E872-82, and E1755-01) [34]. It is worth mentioning that the oxygen (O) and fixed carbon content in elemental and proximate analysis was calculated by difference. In order to improve the overall accuracy of the final results, all measurements were repeated three times, calculating the average values.

### 2.4. Thermogravimetric Measurements

Thermogravimetric analysis (TGA; model: STA449 F3; NETZSCH, Selb, Germany) was used to evaluate the thermal decomposition of the milled samples in nitrogen ambient. The thermal decomposition experiments using TGA were performed on 7–10 mg from each sample until 900 °C with nitrogen flow rate: 60 mL min^−1^. In order to study the effect of heating rate on the thermal decomposition and chemical decomposition of GFRC (in the next section), the TGA experiments were carried out at different heating rates: 5, 10, 15, 20, 25, and 30 °C min^−1^, because several studies showed that the heating rate has a significant effect on the yield of the formulated volatile products. In addition, the intensity of these volatile compounds increased with the increase of the heating rate [34,35]. Meanwhile, the kinetic analysis using linear and nonlinear isolation methods was performed on three different heating rates lower than 20 °C min^−1^, particularly 5, 10, and 15 °C min^−1^ [35]. Finally, the weight loss obtained at each heating rate was recorded versus pyrolysis temperatures using Pyrys software. Then, fitting TGA curves was followed by estimating DTG curves through numerical derivation of the obtained TGA data and then fitting the DTG curves and determining the maximum thermal decomposition peaks and their intensity and position. Finally, the pyrolysis parameters of the decomposed GFRP at heating rates (5–30 °C min^−1^) and their effect on the devolatilisation index (Di) of volatile matters released during the pyrolysis experiments using TGA were determined using Equation (1), where ΔT is defined as the changing in temperature in the range equal Rd/Rmax = 0.5 and Rd is defined as a decomposition rate, and all parameters can be extracted from TGA-DTG curves [36]. In addition, the heat-resistance index (THRI) was determined (using Equation (2)) to measure of the ability of GFRC to resist a heat flow and confirm the thermal stability trend observed using TGA-DTG measurements [37]. It is worth mentioning that T_5_ and T_30_ are defined as the temperatures at 5% and 30% of weight losses, respectively and can be extracted from TGA data.

(1) $$D_{i}=\frac{\mathrm{weight}\;\mathrm{loss}\;\mathrm{rate}\;(R_{\max})}{\mathrm{Initial}\;\mathrm{decomposition}\;\mathrm{temperature}\;\left(T_{i})\times \;\mathrm{Maximum}\;\mathrm{decomposition}\;\mathrm{temperature}\;(T_{m}\right)\times \left(\Delta T\right)}$$

THRI = 0.49 × [T_5_ + 0.6 × (T_30_− T_5_)] (2)

### 2.5. Chemical Analysis of the Formulated Chemical Compounds

The functional groups of the formulated chemical compounds at the maximum decomposition temperatures for each heating rate were examined using TG-FTIR. In order to identify and quantified the chemical compounds of these formulated volatile products correctly, the thermogravimetry–gas chromatography–mass spectrometry (TG–GC–MS, Thermo Scientific ISQ™ single quadrupole GC–MS) was used. The GC–MS measurements were carried out using a laboratory test rig composed of a micro Automation Autoinjector™ unit connected with TGA analyser to collect the formulated gases at the specified temperatures, then analysing their chemical compounds using GC-MS (Shimadzu GC-2010) according to the following conditions: scanning range 30–600 m/s, pumping time (20 s), column setting (Argon ambition with purity ≥99.999%, 20 psi, 90 °C, and 130 s), inject time and temperature (30 ms and 90 °C), and TCD temperature (75 °C at 50 Hz) [38,39].

### 2.6. Pyrolysis Kinetics of the Milled GFRC

The pyrolysis kinetics of the milled GFRP in terms of activation energy (Ea) at each conversion rate was determined using linear isoconversional methods (KAS, FWO, and Friedman) (Equations (3)–(5)) and nonlinear isoconversional methods (Vyazovkin, and Cai) (Equations (7)–(11)), as shown in Table 1. In case of the Vyazovkin method, the activation energy for each conversion rate was calculated through numerical integration of Equation (6) and constrained by minimising Equation (7). In order to solve this integral and $h\left(x\right)$ term, Equations (8) and (9) were used. Regarding the Cai model, the activation energies were determined using Equation (10) and all codes were built using MATLAB software. In contrast, DAEM and IPR models were used to fit the TGA-DTG experimental data using Equations (11) and (12) and to estimate the activation energy and pre-exponential factor. Finally, the deviation (Dev.%) between the calculated and experimental TGA-DTG data were estimated using Equation (13).

## 3. Results and Discussion

### 3.1. Microstructure of the Fabricated GFRC

**Figure 2:**
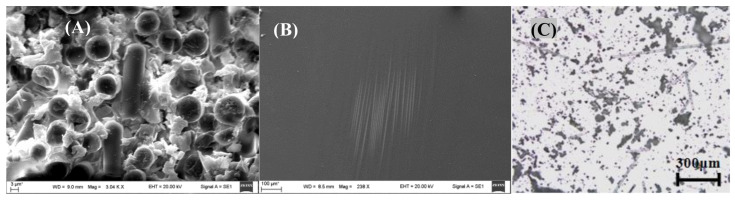
(**A**) SEM micrograph of the fracture cross-section, (**B**) SEM image of the surface of the fabricated GFRC laminates, and (**C**) Metallographic image of the milled GFRC sample.

Figure 2 shows the microstructure cross-section and morphology SEM images of the fabricated panel (after and before crushing). It is clear that the cured panel was composed of many laminates containing destroyed weft (horizontal direction) and warp yarns (vertical direction) resulting from the shear stress produced during cutting the samples. These yarns are joined together using epoxy resin. Figure 2A shows the shape of the warp yarns (vertical direction) that have round shapes with similar size and good distribution and covered with epoxy debris in the form of flakes and bulk particles, which indicate that failure was occurring in the form of ductile fracture [46]. In contrast, the surface morphologies of panel surface are very smooth with little debris produced during the cutting process without any cracks or notched, as shown in Figure 2B, which means that the preparation and curing processes were occurring according to the standard methods [47]. Figure 2C shows the metallurgical image of the milled GFRC powder. As shown, the powder has a high degree of fineness in microscale and is composed of two components: fibreglass threads or particles and epoxy resin separated in the form of fibreglass–epoxy composite agglomerations.

### 3.2. Basic Properties of GFRC

Table 2 shows the ultimate and proximate analyses of GFRC powder. As shown in the table, the ultimate measurements showed that carbon (C) and oxygen (O) are the main elements in the tested GFRP powder with an average amount of 32.67 wt% and 61.24 wt%, respectively. In addition, a small amount of hydrogen (H) was observed with concentration estimated at 3.94 wt%. The strong presence of these elements (C, O, and H) indicates that GFRP can be considered as a promising source of energy products and carbon precursor. In addition, a small percentage of nitrogen (N) was also noticed during the measurements (2.16 wt%) with a completely absence of sulphur (S), which helps to decrease toxic emissions (e.g., SO_2_ and NOx) during the pyrolysis process and their potential application at large scale [48]. In contrast, the proximate measurements showed that the GFRP powder is rich with a volatile matter content (42.28 wt%) that contributes to increase the heating value of the formulated fuel during the conversion process. In addition, a high amount of ash content (55.1 wt%) was observed that can act as a catalyst during the thermal decomposition, which leads to change reactivity of the feedstock (GFRC powder) to be more activated and increased yield and quality of the obtained oil [49].

### 3.3. TG-DTG Data Analysis

**Figure 3:**
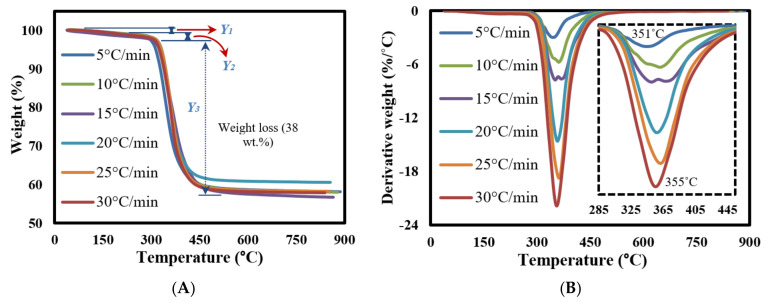
(**A**) TGA and (**B**) DTG curves of FGE and GFRC at different heating rates.

Figure 3 shows the TGA-DTG curves of the milled GFRC for all tested heating rates. It seems that the milled samples are decomposed in successive three stages as shown in the TGA analysis curves (Figure 3A) with almost similar features even with changing heating rates, but with different weight loss and position of decomposition zones. As shown, the first weight loss is located in the range of 50–200 °C (Y1) due to evaporation moisture with a very small weight loss <1 wt%. After that, another weak decomposition zone (Y2) was observed due to decomposing some organic components in epoxy and hardener [50], while the main degradation stage (Y3) was located in the range of 317–467 °C with a weight loss of 38 wt%. This degradation zone is related to the full degradation of epoxy and its compounds, especially the bromine element [51]. In the last decomposition stage, a significant decomposition was observed (42–46%) due to the formulated char mixed with the fibreglass particles, which needs a very high temperature for decomposition them, and that is why it remained a solid residue [52]. Actually, these results agree with the preparation matrix conditions and concentrations, which showed the amount of epoxy estimated at 38%, which means that all the epoxy fraction was decomposed and fibreglass remained mixed with char.

Finally, DTG curves showed that all the feedstock components are decomposed together in a major single peak reaction (since the weight loss of other stages was very small). It seems that all samples had similar weight loss, which indicate that the heating rates did not have an effect on the weight loss and the main changes were happening in the chemical structure of the formulated components as shown in the next sections. In addition, it was observed that the maximum decomposition temperature increased gradually from 400 to 430 °C by increasing the heating rates as a result of increasing the applied temperature on the surface are of the milled samples, which led to increasing the generated heat fluxes, facilitating their transmission inside the internal molecular and accelerating the decomposition reaction in less time until decomposing the feedstock completely [34]. All the pyrolysis characteristic parameters are shown in Table 3.

### 3.4. Chemical Analysis of the Synthesised Chemical Compounds

**Figure 4:**
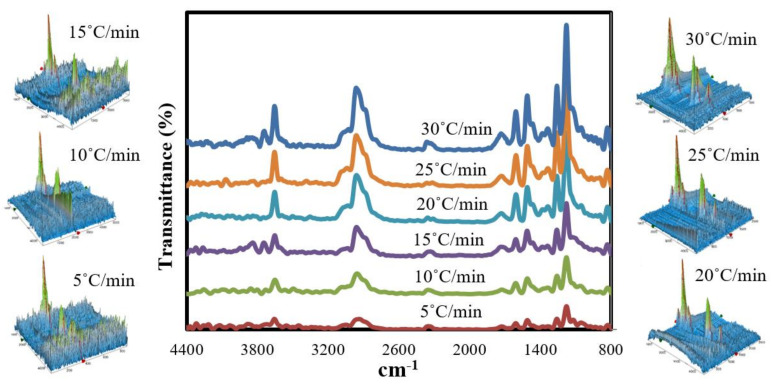
Two-dimensional (2D)-3D FTIR analysis of the decomposed milled GFRP at different heating rates.

FTIR coupled with TG was used to examine the obtained chemical compounds at the maximum decomposition temperatures (based on the DTG results) of GFRC for all the specified heating rate ranges. The FTIR measurements were performed in the ranges from 346 °C to 364 °C, and all results are shown in Figure 4. As shown in the 2D FTIR spectra, the tested GFRC samples under the specified heating rates give almost the same features in terms of the generated functional groups, particularly sharp peaks in the range 1200–1250 cm^−1^ assigned to aromatic benzene compound, 1300 and 1500 cm^−1^ related to the N-O group, 1700 cm^−1^ being the typical place for carbonyl (C=O), 2300 cm^−1^ being the typical place of CO_2_, and 3600 cm^−1^ due to an aromatic and aliphatic C-H group. It seems that the decomposed samples are rich with aromatic compounds and the absorbances of these compounds increased significantly as the heating rate increased as a result of the abundance in the produced heat flux, which led to decomposition of the whole complex of organic molecules into aromatic compounds [53]. Meanwhile, the 3D FTIR spectra showed that the disturbance peaks disappeared as the heating rates increased, which was proof that all the organic components in epoxy resin were decomposed to energy and chemical compounds. GC-MS measurement was used in the next section to determine the main composition and yield of these formulated compounds.

### 3.5. Chemical Analysis of the Synthesised Chemical Compounds Using GC–MS

**Figure 5:**
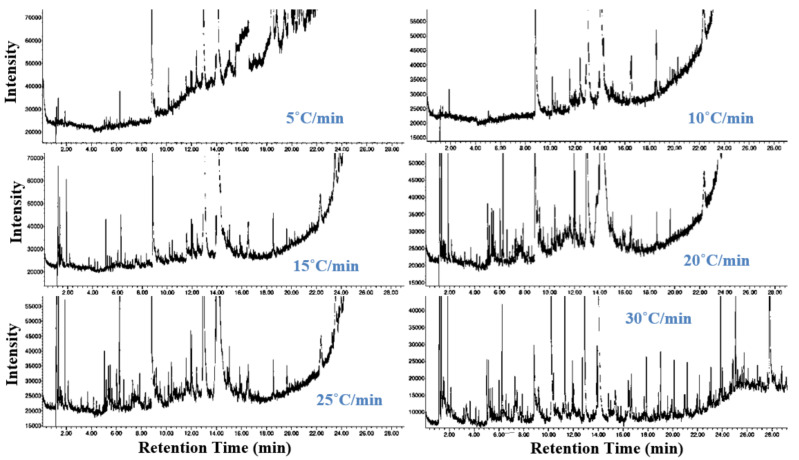
GC-MS analysis of the decomposed GFRC at different heating rates.

Figure 5 shows the GC–MS results of the generated chemical compounds at maximum decomposition zones of GFRP samples under the different heating rates, and the obtained chemical compounds with their respective peak areas are shown in Table 4. According to GC-MS analysis, the synthesised volatile products are composed of many chemical compounds. Phenol (4.25–26.99%), phenol, 4-(1-methylethyl) (10.31–40.08%), and p-Isopropenylphenol (23.64–34.21%) were represented as the major compounds in GC-MS analysis, and these compounds can be separated using membranes or other technologies [54,55]. As shown, the yield of these compounds changed with increase of the heating rate due to the significant increase in the produced heat flux during the process. The strong presence of phenol and p-isopropenylphenol compounds confirms that the formulated products from the pyrolysis of GFRC were typical energy products [56,57]. In addition, these pyrolysis compounds (phenol, p-isopropenylphenol, etc.) can be used in many fields such as chemicals, fuel, pharmaceuticals, etc. Based on these results, the pyrolysis process is recommended to convert GFRC into high added energy products and the recovery of short glass fibres, especially at high heating rates.

### 3.6. Kinetic Analysis of FEC Pyrolysis

#### 3.6.1. Estimation of Activation Energies Using Isoconversional Methods

**Figure 6:**
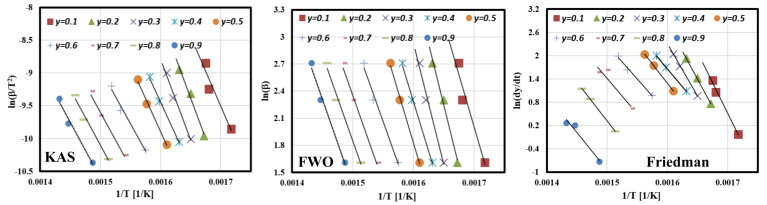
Plots of isoconversional methods at different values of conversion.

**Figure 7:**
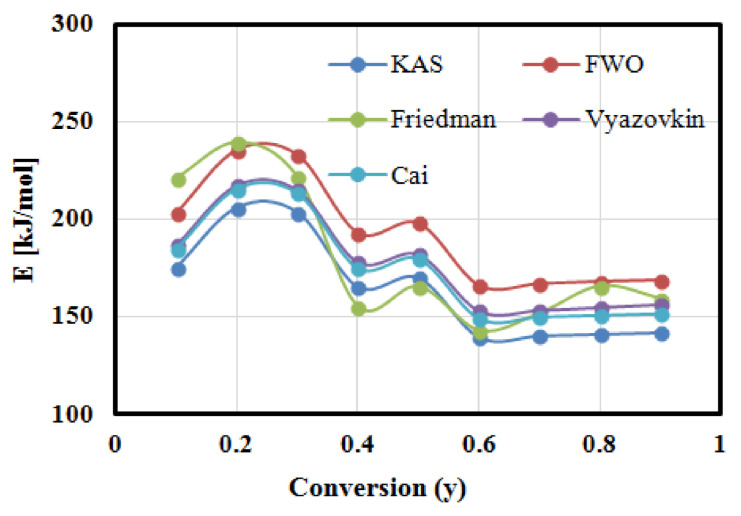
The relationship between apparent activation energy and conversion rates.

Figure 6 shows KAS, FWO, and Friedman fitting curves for conversion rate from 10% to 90% at heating rates: 5, 10, and 15 °C min^−1^. The activation energies can be calculated from the slope of these plotting curves, which are represented by −Ea/R (KAS and Friedman) and −1.0516Ea/R (FWO). As shown from the fitting curves, KAS and FWO plots are composed of parallel straight lines in the main conversion range (20–80%). Meanwhile, the Friedman plot is composed of straight lines distributed randomly with a big variation between the fitting points, which means that FWO and KAS models are valid techniques to model the reaction mechanism of GFRC pyrolysis in the major conversion phase, and the values of the calculated Ea at all conversion rates are summarised in Figure 7 and Table 5. In addition, the coefficient of determination (R^2^) was calculated to evaluate the accuracy of the fitted curves as shown in Table 5. As shown, the average Ea is estimated at 164.5886 kJ mol^−1^ (KAS), 192.6161 kJ mol^−1^ (FWO), and 180.3665 kJ mol^−1^ (Friedman). It is clear that all models gave much less error with R2 in the ranges 0.9804-0.9918. As mentioned in the Introduction section, several studies have been developed to determine the Ea of pyrolysis of GFRC and were estimated at 28.17 kJ/mol (GFRP waste was a by-product from the acid solution storage tank manufacturing factory) and 41.4–78.4 kJ/mol (for by-product from reuse plastic fuel) [26,27], which means that the GFRC (fibreglass and epoxy) produced an Ea higher than other types of GFRC by 78% and 52%, respectively. This is due to the fact that the GFRC used in the current research was composed of two mainly components, including glass fibre and epoxy, and each component has a different Ea. However, the glass fibre did not decompose alongisde epoxy in a single reaction (based on the DTG results), and the obtained Ea represents the summation of Ea of all the decomposition of the epoxy component only. This is why the pyrolysis of GFRC gave a higher Ea value compared to other feedstocks, and it is supposed to compare with pure epoxy, which was estimated at 130 to 230, 78–262, and 76.2–327 kJ/mol [58]. This difference in results is due to the different chemical composition, size, crystallinity, and testing ambient, which led to the digestibility of GFRC and conversion into energy products [59,60]. In order to improve the accuracy of these results, nonlinear isoconversional methods were used in the next section.

#### 3.6.2. Estimation of Activation Energies Using Nonlinear Isoconversional Methods

**Figure 8:**
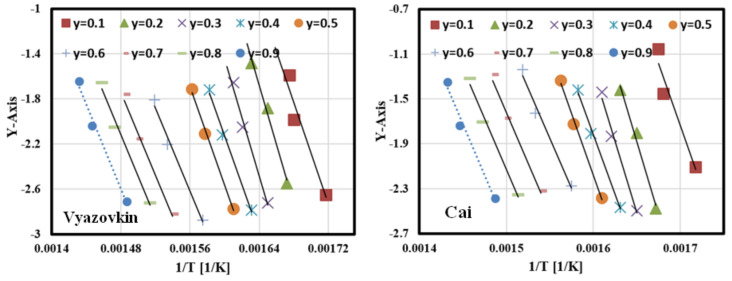
Plots of nonlinear isoconversional methods at different values of conversion.

The activation energy at each conversion rate was determined numerically using nonlinear isoconversional methods (Vyazovkin and Cai approaches) after several iterations at 5, 10, and 15 °C min^−1^. The solution started with Ea = 200 kJ/mol (as an initial condition) until the values of Ea became constant; then, it took these values as optimal values of Ea and all iterations, as shown in Table 6 and Table 7. As shown in the tables, Ea values became constant in both cases at the third iteration. These values were used to plot Vyazovkin and Cai curves at all conversion rates as shown in Figure 8, where the *Y*-axis in the case of Vyazovkin is represented by Equation (14), while Equation (15) represents the *Y*-axis in case of Cai methods [44,45]. It is clear that the determined Ea values using nonlinear isoconversional methods were fully matched with those calculated by linear isoconversional methods with R^2^ >98, which means that linear and nonlinear isoconversional methods are valid for simulating the pyrolysis kinetics of GFRC.

(14) $$\ln\left\{\frac{\beta_{i}}{T_{y,i}^{2} [h\left(x_{y,i}\right)-\frac{x_{y,i}^{2}e^{x_{\alpha ,i}}}{x_{y,i-0.1}^{2}e^{x_{y,i-0.1}}}h\left(x_{y,i-0.1}\right)]}\right\}$$

(15) $$\ln\left\{\frac{\beta_{i}}{T_{\alpha ,i}^{2} [h\left(x_{\alpha ,i}\right)-\frac{x_{\alpha ,i}^{2}e^{x_{\alpha ,i}}}{x_{\alpha -\Delta \alpha ,i}^{2}e^{x_{\alpha }-\Delta \alpha ,i}}h\left(x_{\alpha -\Delta \alpha ,i}\right)]}\right\}$$

#### 3.6.3. Fitting of TGA-DTG Data Using DAEM and IPR

**Figure 9:**
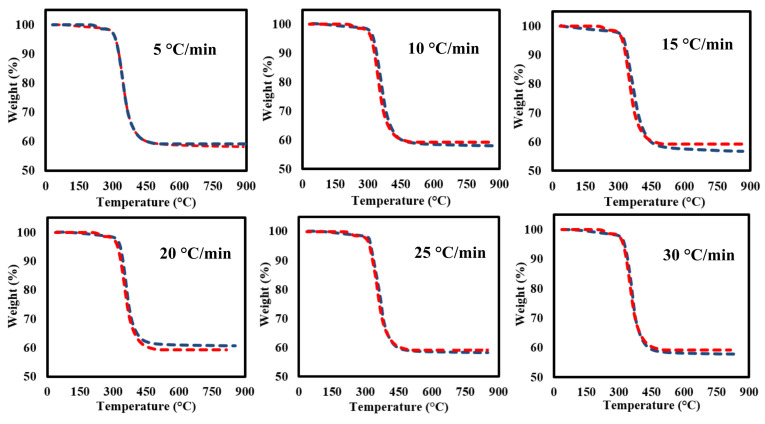
TGA experimental and calculated data at 5–30 °C/min.

**Figure 10:**
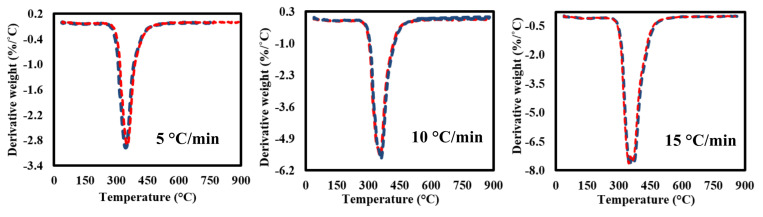

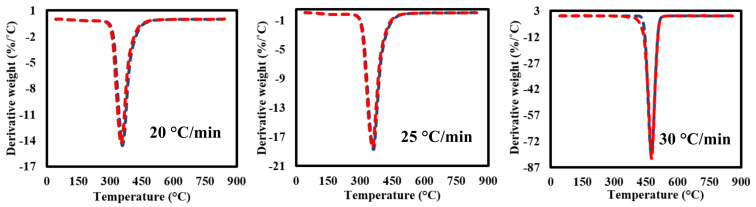
DTG experimental and calculated data at 5–30 °C/min.

Figure 9; Figure 10 shows the TGA-DTG experimental data (red dotted lines) and fitting (blue dotted lines) curves of TGA-DTG at 5–30 °C/min for GFRC sample using Equations (11) and (12). It seems that the plotting TGA-DTG data fully matched with the experimental curves with a deviation less than <1 for all heating rates in both cases DAEM (TGA curves) and IPR (DTG curves), indicating that the DAEM and IPR models succeeded in the fitting of thermal decomposition curves of GFRC pyrolysis. Based on the DAEM and IPR models, activation energy and pre-exponential factors were determined, and all values are summarised in Table 8. According to the TG-FTIR-GC-MS analysis of GFRC, the pyrolysis process can be considered as a promising approach to dispose of GFRC and convert them into energy components and fibre powder. In addition, nonlinear isoconversional methods were successful to determine kinetic parameters and to plot TGA-DTG curves of GFRC.

## 4. Conclusions

In this research, the basic fundamentals of pyrolysis treatment fibreglass/epoxy composites (GFRC) using the TG-FTIR-GC-MS measurements were studied. The pyrolysis kinetics of GFRC were also investigated using linear and nonlinear isoconversional methods, including KAS, FWO, Friedman, Vyazovkin, and Cai models. The research started with a production of the GFRC panel; then, it analysed the element and ultimate properties, which was followed by studying the thermal and chemical decomposition properties of GFRC and determining the chemical compounds using the TG-FTIR-GC-MS system. Based on the obtained TGA-DTG curves, the activation energies for all the processes and for each conversion rate was calculated using linear and nonlinear isoconversional methods, followed by modeling TGA and DTG curves using DAEM and IPR models. The measurements and results of pyrolysis GFRC revealed the following:

(A) The TGA-DTG results showed that GFRC decomposed thermally in three phases with a total mass loss of 43% and the major decomposition region was located in the range of 256–500 °C.
(B) TG-FTIR spectra showed that the aromatic benzene and C-H bond were the main volatile compounds in the decomposed samples, and its abundance increased with the increase of heating rate.
(C) GC-MS results showed that phenol (4.25–26.99%), phenol, 4-(1-methylethyl)- (10.31–40.08), and p-isopropenylphenol (23.64–34.21%) were the main volatile and flammable compounds, and their yield was affected sigifcantly by the heating rate.
(D) The kinetic models using linear and nonlinear isoconversional methods revealed that the average activation energies can be estimated at 165 KJ/mol (KAS), 193 KJ/mol (FWO), 180 KJ/mol (Friedman), 177 KJ/mol (Vyazovkin), and 174 KJ/mol (Cai) with R^2^ >98.
(E) DAEM and IPR models showed a high performance for plotting of the TGA-DTG experimental data of GFRC samples for all heating rates with deviation lower than <1 for TGA and DTG curves.

Based on the advertised results, the pyrolysis treatment can be employed as a cleaner and sustainable technology for converting epoxy to energy products. In addition, the linear and nonlinear isoconversional methods can be used to model the pyrolysis kinetics of GFRC under applying any heating rate in the main conversion zone (20–80%). The DAEM and IPR models are also highly recommended for plotting the TGA-DTG curves of GFRC with high predictability. In addition, GFRC can be used as a promising new source of renewable energy.

## Figures and Tables

**Table 1:** Linear and nonlinear isoconversional methods used to determine kinetic parameters for the pyrolysis of GFRC [40,41,42,43,44,45].

Equation No.	Method	Expressions	Plots	Slope Value
(3)	Kissinger–Akahira–Sunose	$\ln\left(\frac{\beta }{T^{2}}\right)$ $=\;\ln\left(\frac{\mathrm{AR}}{\mathrm{Eag}\left(y\right)}\right)\;$ $-\frac{-\mathrm{Ea}}{\mathrm{RT}}$	ln(β/T^2^) versus 1/T	−Ea/R
(4)	Flynn–Wall–Ozawa	lnβ $=\left(\frac{\mathrm{lnAEa}}{\mathrm{Rgy}}\right)\;$ $-5.335-\frac{1.0516\mathrm{Ea}}{\mathrm{RT}}$	lnβ versus 1/T	−1.0516Ea/R
(5)	Friedman	$\ln\left(\frac{\beta \mathrm{dy}}{\mathrm{dT}}\right)$ $=\ln\left(\mathrm{Af}\left(y\right)\right)\left(\frac{-\mathrm{Ea}}{\mathrm{RT}}\right)$	ln(dy/dt) versus 1/T	−Ea/R
(6)	Vyazovkin	$\left(\alpha \right)\;=\int_{0}^{\alpha }\frac{\mathrm{dy}}{f\left(y\right)}=A\int_{0}^{t}\exp\left(\;,-E/\mathrm{RT}\right)\mathrm{dt}$		−Ea/R
(7)		$\Phi \left(E_{y}\right)=\sum_{i=1}^{n}\sum_{j\neq i}^{n}\frac{I{\left(E_{y}\;,{\;T}_{y,i}\right)}_{j\beta }}{I{\left(E_{y}\;,T_{y,j}\right)}_{i\beta }}$		
(8)		$\{I\left(E,T\right)=\int_{0}^{T}e^{-E/\mathrm{RT}}\mathrm{dT}=\frac{{\mathrm{RT}}^{2}}{E}e^{-E/\mathrm{RT}}\;h\left(x\right)$}		
(9)		$h\left(x\right)=\frac{x^{4}+18x^{3}+86x^{2}+96x}{x^{4}+20x^{3}+120x^{2}+240x+120}$		
(10)	Cai	$\ln\left\{\frac{\beta_{i}}{T_{y,i}^{2} [h\left(x_{y,i}\right)-\frac{x_{y,i}^{2}e^{x_{y,i}}}{x_{y-\Delta y,i}^{2}e^{x_{y-}\Delta y,i}}h\left(x_{y-\Delta y,i}\right)]}\right\}=\ln\;\left\{\frac{A_{y-\Delta y/2}R}{E_{y-\Delta y/2}\;g\left(y,y-\Delta y\right)}\right\}-\frac{E_{y-\Delta y/2}}{{\mathrm{RT}}_{y,i}}$		−Ea/R
(11)	DAEM	$\ln\left(\frac{\beta }{T^{2}}\right)$ $=\;\ln\left(\frac{\mathrm{AR}}{\mathrm{Ea}}\right)+0.6075\;$ $-\frac{\mathrm{Ea}}{\mathrm{RT}}$		
(12)	IPR	${\frac{\mathrm{dm}}{\mathrm{dt}}}^{\mathrm{calc}}=-\left(m_{0}-m\right)\sum_{i=1}^{3}.C_{i}\frac{{\mathrm{dX}}_{i}}{\mathrm{dt}}$		
(13)		Dev. $(\%)=\frac{100\sqrt{F.O._{\mathrm{DTG}}\left(Z-N\right)}}{\max\left(\left|\mathrm{dm}/\mathrm{dt}\right|\right)}$		

**Table 2:** Ultimate and proximate analysis of the milled GFRP.

Elemental Analysis (wt%)					Proximate Analysis (wt%)			
N	C	H	S	O	Moisture	Volatile Matter	Fixed Carbon	Ash
2.16 ± 0.09	32.67± 0.23	3.94± 0.06	<0.01± 0.00	61.24 ± 0.26	0.08 ± 0.00	42.28 ± 0.13	2.54 ± 0.07	55.1 ± 0.18

**Table 3:** The pyrolysis characteristic parameters for GFRC at different heating rates.

Pyrolysis Parameters	Heating Rate (°C/min)					
	5	10	15	20	25	30
Onset temperature Ti (°C)	256	260	278	308	286	279
Tm (°C)	346	361	367	357	364	355
Tf (°C)	477	478	500	462	454	466
Rmax (%/min)	2.9	5.8	7.8	14.6	18.7	21.9
Di (% min^−1^ °C^−3^)	5.2 ×10^−7^	8.7 ×10^−7^	9.8 ×10^−7^	2.2 ×10^−6^	2.9 ×10^−6^	3.9 ×10^−6^
ΔT	63	71	78	60	62	57
Mf (%)	58.2	58.03	56.69	60.59	58.25	57.87
T5	301.4	310.8	314.9	319.2	317	316.6
T30	364.5	377.1	384.6	380	394.9	393.5
THRI	166.24	171.78	174.79	174.28	178.23	177.74

**Table 4:** GC-MS compounds generated at 5–30 °C/min.

5 °C/min			10 °C/min			15 °C/min			20 °C/min			25 °C/min			30 °C/min		
Time (min.)	GC Compounds	Area (%)	Time (min.)	GC Compounds	Area (%)	Time (min.)	GC Compounds	Area (%)	Time (min.)	GC Compounds	Area (%)	Time (min.)	GC Compounds	Area (%)	Time (min.)	GC Compounds	Area (%)
1.219	Methane, chloro-	1.99	1.219	Methane, chloro-	2.26	1.226	Methane, chloro-	5.34	1.200	Propene	2.83	1.219	Methane, chloro-	6.26	1.200	Methane, chloro-	7.55
8.794	Phenol	26.99	8.807	Phenol	15.25	1.368	2-Hexanone, 4-methyl-	2.09	1.362	Acetone	2.68	1.362	Acetic acid, sodium salt	3.40	1.349	Acetic acid, sodium salt	4.64
10.159	Phenol, 3-methyl-	1.51	10.165	Phenol, 4-methyl-	2.00	1.892	Furan, tetrahydro-	2.21	1.886	Furan, tetrahydro-	2.17	1.886	Furan, tetrahydro-	1.96	1.866	Furan, tetrahydro-	2.77
12.397	2-Allylphenol	1.68	11.563	Phenol, 2-ethyl-	2.18	5.062	1,3-Dioxol-2-one,4,5-dimethyl-	1.67	5.055	Piperazine, 1,4-dimethyl-	1.65	5.055	Piperazine, 1,4-dimethyl-	1.60	6.239	Benzene, 1,3-dimethyl-	3.48
12.928	Phenol, 4-(1-methylethyl)-	10.31	12.397	2-Allylphenol	2.47	6.278	p-Xylene	1.88	5.379	1,3-Cyclopentadiene, 5-(1,1-dimethylethyl)-	1.74	5.508	1H-Pyrrole-2-ethanamine, 1-methyl-	1.51	8.820	Phenol	4.25
14.021	p-Isopropenylphenol	24.91	12.921	Phenol, 4-(1-methylethyl)-	40.08	8.820	Phenol	6.89	6.271	p-Xylene	4.40	6.271	p-Xylene	3.96	10.211	4(1H)-Pyrimidinone	9.04
18.420	Silane, [[4-[1,2-bis[(trimethylsilyl)oxy]ethyl]-1,2-phenylene]bis(oxy)]bis[trimethyl-	7.53	14.040	p-Isopropenylphenol	23.64	12.035	2(5H)-Furanone, 4-methyl-3-(2-methyl-2-propenyl)-	1.43	8.813	Phenol	15.11	8.826	Phenol	5.00	11.304	Octahydro-2(1H)-quinolinone	4.06
20.108	N-[5-(3-Hydroxy-2-methylpropenyl)-1,3,4,5-tetrahydrobenzo[cd]indol-3-yl]-N-methylacetamide	3.61	18.536	Silane, [[4-[1,2-bis[(trimethylsilyl)oxy]ethyl]-1,2-phenylene]bis(oxy)]bis[trimethyl-	2.76	12.921	Phenol, 4-(1-methylethyl)-	31.74	11.951	2-Cyclopenten-1-one, 3-methyl-	2.41	10.178	Phenol, 3-methyl-	1.41	12.895	Phenol, 4-(1-methylethyl)-	13.16
20.257	2-Ethylacridine	4.93	23.898	1,1′-Biphenyl, 4-phenoxy-	4.19	13.937	Phenol, p-tert-butyl-	1.92	12.035	Cyclohexanone, 2-methyl-5-(1-methylethenyl)-	1.90	11.951	Cyclohexene, 1-pentyl-	1.35	13.898	Phenol, 2-methyl-5-(1-methylethyl)	2.51
21.647	Cyclotrisiloxane, hexamethyl-	3.13	26.130	Cyclotrisiloxane, hexamethyl-	5.18	14.021	p-Isopropenylphenol	34.21	12.928	Phenol, 4-(1-methylethyl)-	18.79	12.041	trans-4a-Methyl-decahydronaphthalene	1.53	13.989	p-Isopropenylphenol	29.02
21.809	Octasiloxane, 1,1,3,3,5,5,7,7,9,9,11,11,13,13,15,15-hexadecamethyl-	2.49				16.537	Pentasiloxane, dodecamethyl-	2.51	14.027	p-Isopropenylphenol	33.42	12.921	Phenol, 4-(1-methylethyl)-	32.63	23.853	1,1′-Biphenyl, 4-phenoxy-	5.05
23.058	1,2-Benzisothiazol-3-amine tbdms	3.31				18.536	trans-4′-Methyl-4-(methylthio)chalcone	1.51	23.918	1,1′-Biphenyl, 4-phenoxy-	2.69	13.937	Phenol, 2-methyl-5-(1-methylethyl)	2.72	25.069	Piperonal, 6-(4-methoxy-1-cyclohexen-1-yl)-	3.61
23.226	Silane, 1,4-phenylenebis[trimethyl	2.42				23.898	1,1′-Biphenyl, 4-phenoxy-	3.03	25.800	1H-Indole, 5-methyl-2-phenyl-	6.46	14.027	p-Isopropenylphenol	30.29	27.780	Phosphine oxide, diphenylpropenyl-	10.86
23.892	1,1′-Biphenyl, 4-phenoxy-	2.22				26.130	Cyclohexane, 1-ethyl-2-propyl-	3.56	26.136	Cyclooctane, 1-methyl-3-propyl-	3.74	23.918	1,1′-Biphenyl, 4-phenoxy-	2.69			
24.358	Silane, 1,4-phenylenebis[trimethyl	2.95										26.136	Cyclotrisiloxane, hexamethyl-	3.69			

**Table 5:** The estimated activation energy at different conversion rates.

y	KAS (KJ/mol)	R^2^	FWO (KJ/mol)	R^2^	Friedman (KJ/mol)	R^2^	Vyazovkin (KJ/mol)	R^2^	Cai (KJ/mol)	R^2^
**0.1**	175	0.9355	203	0.9381	221	0.9916	186	0.9368	184	0.9325
**0.2**	205	0.9871	235	0.9891	240	0.9992	217	0.9902	215	0.9882
**0.3**	203	0.9835	233	0.9861	222	0.999	215	0.9857	213	0.985
**0.4**	165	0.9945	193	0.9955	154	0.9992	177	0.995	174	0.9951
**0.5**	169	0.9946	198	0.9956	165	0.9999	181	0.9951	179	0.9951
**0.6**	139	0.9822	165	0.9861	142	0.9895	152	0.986	148	0.9844
**0.7**	140	0.9822	167	0.986	151	0.987	153	0.9856	149	0.9844
**0.8**	140	0.9821	168	0.986	165	0.9996	154	0.9854	150	0.9843
**0.9**	141	0.9821	168	0.986	159	0.9613	156	0.9853	151	0.9843
**Avg.**	164	0.9804	192	0.9831	180	0.9918	177	0.9827	174	0.9814

**Table 6:** The determined activation energy using the Vyazovkin method at different number of iterations.

Conversion (y)	The Activation Energy (kJ/mol)				
	Intial Value	First Iteration	Second Iteration	Third Iteration	Fourth Iteration
0.1	200	185.7325984	186.3112627	186.665928	186.665928
0.2	200	216.6053071	217.2801578	217.693776	217.693776
0.3	200	213.9912192	214.6579256	215.066552	215.066552
0.4	200	176.9886399	177.5400617	177.87803	177.87803
0.5	200	180.934589	181.4983048	181.843808	181.843808
0.6	200	151.9479942	152.4214001	152.711552	152.711552
0.7	200	152.4360676	152.9109941	153.202078	153.202078
0.8	200	153.867198	154.3465832	154.6404	154.6404
0.9	200	155.3727803	155.8568563	156.153548	156.153548
**Average**	200	176.4307104	176.980394	177.3172969	177.3172969

**Table 7:** The calculated activation energy using the Cai method at different number of iterations.

Conversion (y)	The Activation Energy (kJ/mol)				
	Initial Value	First Iteration	Second Iteration	Third Iteration	Fourth Iteration
0.1	200	184.720452	184.712138	184.712138	184.712138
0.2	200	215.557078	214.675794	215.62359	215.62359
0.3	200	213.154332	212.289676	213.220844	213.220844
0.4	200	174.751966	173.945508	174.80185	174.80185
0.5	200	179.4768122	178.751	179.49926	179.49926
0.6	200	148.90374	148.28019	148.895426	148.895426
0.7	200	149.851536	149.252928	149.843222	149.843222
0.8	200	150.64968	150.059386	150.641366	150.641366
0.9	200	151.506022	150.94067	151.497708	151.497708
**Average**	200	174.2857354	173.6563656	174.3039338	174.3039338

**Table 8:** The calculated DAEM and IPR parameters.

	DAEM	IPR
E1	200.382	10.857
A1	3.33 × 10^20^	5.55 × 10^14^
E2	248.47	234.67
A2	3.66 × 10^20^	1.40 × 10^17^

## Data Availability

Not applicable.
